# Comparison of euploidy rates between progestin-primed ovarian stimulation and GnRH antagonist protocols: a single-center study in a mixed-ethnicity population

**DOI:** 10.3389/fendo.2026.1788942

**Published:** 2026-05-07

**Authors:** Caroline Ingold, Giovanna Giovacchini dos Santos, Caio Parente Barbosa, Renato De Oliveira

**Affiliations:** Instituto Ideia Fertil, Centro Universitário FMABC, Santo André, SP, Brazil

**Keywords:** aneuploidy, assisted reproductive techniques, ovulation induction, preimplantation diagnosis, progestins

## Abstract

**Objective:**

To compare the euploidy rate between ovulation suppression protocols using PPOS and GnRH-ant, and to evaluate possible variables associated with euploidy rate such as age, BMI, infertility duration, treatment-related factors, and semen parameters, in a genetically admixed Brazilian population underrepresented in reproductive medicine research.

**Design:**

This was a retrospective non-inferiority case-control study including 268 IVF cycles, conducted between 2021 and 2022. Subjects: A total of 268 *in vitro* fertilization (IVF) cycles were included, all with indication for preimplantation genetic testing for aneuploidy (PGT-A), of which 64.5% (n = 173) were included in the PPOS group, and 35.5% (n = 95) in the GnRH-ant group. A statistical power of 85% was achieved for the primary outcome.

**Exposure:**

Patients underwent controlled ovarian stimulation using either progestin-primed ovarian stimulation (PPOS) or GnRH antagonist (GnRH-ant) protocols.

**Main outcome measures:**

The primary outcome was the euploidy rate in PPOS and GnRH-ant groups.

**Results:**

No significant differences were observed in euploidy rate between the PPOS and GnRH-ant groups (25.9% vs. 27.5%; p = 0.865; r = 0.01). In the adjusted analysis, stimulation protocol was not significantly associated with euploidy rate (coefficient = 3.38; 95% CI: -14.26 to 16.03), whereas maternal age remained significantly and negatively associated with the outcome (coefficient = -7.83; 95% CI: -11.85 to -3.81). Fertilization rate was higher in the GnRH-ant group (p = 0.004), but this did not affect embryo formation or euploidy outcomes. Paternal age and semen parameters were not significantly associated with euploidy.

**Conclusion:**

According to the prespecified criterion, PPOS met the formal criterion for non-inferiority in relation to GnRH-ant regarding euploidy rate, although with limited margin. These findings support PPOS as a viable option in freeze-all/PGT-A cycles, but they should be interpreted with caution given the retrospective design and baseline differences between groups.

## Introduction

Ovulation suppression is essential during controlled ovarian stimulation (COS). Although GnRH agonists were historically used, their side effects and OHSS risk led to the adoption of GnRH antagonists as a safer alternative, although at higher cost. With advances in embryo vitrification, progestin-primed ovarian stimulation (PPOS) emerged as an effective strategy to prevent the LH surge through progestin-mediated hypothalamic–pituitary suppression (1–4).

Compared to daily subcutaneous injections of GnRH-ant, PPOS may offer greater convenience because of oral administration and has been associated with lower medication costs in previous reports (5, 6). Treatment cost is an important limiting factor for many couples undergoing assisted reproduction (5). According to La Marca et al., the cost of GnRH-ant ranges from €190 to €320 per cycle, whereas oral progestins used in PPOS cost approximately €10 to €15.6 (6).

The endometrial impairment associated with PPOS – which typically precludes fresh embryo transfer – becomes less relevant in specific scenarios, such as fertility preservation for age-related or oncological reasons (7, 8), high-risk patients for OHSS (9), oocyte donors, and those undergoing preimplantation genetic testing for aneuploidy (PGT-A) (10). In these cases, fresh embryo transfer is not performed, making PPOS a potentially useful alternative in these settings (11).

With the increasing use of PPOS, concerns have emerged regarding embryo quality in patients undergoing COS (12). In 2019, La Marca et al. analyzed euploidy rates in patients treated with PPOS compared to conventional protocols and found similar rates between the two groups, encouraging the use of this protocol (3). It is noteworthy that most studies on PPOS have been conducted by its original proponents and in populations with lower genetic diversity.

The objective of this study is to compare two COS protocols used in assisted human reproduction – ovulation suppression using GnRH-ant and PPOS – to verify non-inferiority between them regarding euploidy rates, in a highly admixed population, with unique genomic characteristics, that may influence treatment responses (13–16). Although ethnic diversity is not assumed to directly determine embryo aneuploidy, evaluating ovarian stimulation strategies in underrepresented populations contributes to assess the external validity and generalizability of existing evidence.

Thus, this study may also contribute to a better understanding of reproductive treatment outcomes and to validate the use of PPOS in groups still underrepresented in scientific literature (14). The establishment of the euploidy rate is justified as it serves an objective parameter to evaluate the impact of the ovulation suppression protocol on embryo quality and is less susceptible to external variables than the pregnancy rate – such as uterine and immunological influences on implantation and gestation (17).

## Materials and methods

This is a retrospective non-inferiority case-control study derived from electronic medical records of the Human Reproduction Department at Instituto Ideia Fértil. It included patients who underwent COS with PPOS and GnRH-ant between 2021 and 2022, all of whom were indicated for PGT-A. The STROBE (Strengthening the Reporting of Observational Studies in Epidemiology) guidelines were followed (18).

The primary variables are the COS protocols (PPOS and GnRH-ant). The primary outcome is euploidy. Secondary variables included age and body mass index (BMI) (19), duration of infertility (20), sperm concentration per milliliter, and sperm morphology according to Kruger’s criteria. Regarding treatment, the variables considered were: antral follicle count (AFC), number of follicles larger than 14 mm at the end of COS, number of oocytes retrieved, number of oocytes in metaphase I and II (MI and MII, respectively), number of fertilized oocytes, fertilization rate of MII oocytes, number of blastocysts formed, number of blastocysts evaluated, number of blastocysts formed on day 5 (D5) and day 6 (D6). The number of treatment days and total medication dose used were also assessed (21–23).

Data were collected on comorbidities, obstetric history, smoking, illicit drug use, and causes of infertility, including endometriosis (ASRM classification, 1996) (24), unexplained infertility, premature ovarian insufficiency (POI), implantation failure, uterine and tuboperitoneal factors, Müllerian malformations, polycystic ovary syndrome (PCOS) (25), and genetic abnormalities. For the male factor, medical records included karyotype results, presence of microdeletions, prior vasectomy, varicocele, hormonal alterations, and semen parameters according to the 6th edition of the World Health Organization Manual (26).

Regarding embryo analysis, embryos were evaluated based on their respective PGT-A results and classified as either euploid or aneuploid. In the present study, the aneuploid category included embryos identified as mosaic, regardless of the degree of mosaicism, because this reflected the clinical practice adopted at our center during the study period (2021–2022), when mosaic embryos were generally managed as non-euploid for clinical decision-making (10). This classification was used for the primary analysis and should be interpreted within the temporal and clinical context of the study period.

Inclusion criteria were patients who underwent treatment using doses of 100 IU to 200 IU of rFSH, with the use of either PPOS or GnRH-ant, and indications for PGT-A such as: maternal age >35 years, recurrent spontaneous miscarriage (≥2), abnormal semen analysis, and couples diagnosed with implantation failure (defined as four transfers of good-quality embryos, with at least three IVF cycles including fresh or frozen embryo transfers, in women under 40 years old, and with recurrent pregnancy loss (27)) (28, 29).

Exclusion criteria included missing information in medical records for any of the previously mentioned variables, patients with canceled cycles, a history of chemotherapy or radiotherapy, and couples who used donor gametes.

The control group underwent COS with hypothalamic suppression using GnRH-ant (Orgalutran^®^ 0.25 mg or Cetrotide^®^ 0.25 mg), initiated when the leading follicle reached 14 mm in diameter, characterizing the flexible protocol (29–32).

The PPOS group underwent COS with hypothalamic blockade using 2 tablets of dydrogesterone (Duphaston^®^ 10 mg; Abbott) once daily, administered orally starting from the day of initiation of exogenous gonadotropins (29). This was a retrospective non-inferiority case-control study based on electronic medical records. As expected in a real-world retrospective cohort, allocation to PPOS or GnRH-ant was not randomized and reflected routine clinical decision-making, including physician judgment and medication availability (33, 34).

This study includes all patients who met the selection criteria between the years 2021 and 2022. Participant characteristics are presented descriptively (minimum and maximum values, absolute numbers, percentages, medians, and standard deviations). For the sample size calculation, non-inferiority, assuming a 15% difference, 178 participants are required – 89 per group (35).

This project was approved by the Research Ethics Committee of the ABC Medical School (CAAE: 90584718.8.0000.0082).

### Statistical analysis

Sample characterization was performed using absolute and relative frequencies of clinical, reproductive, and embryonic variables. Comparisons between the PPOS and GnRH-ant groups were made using Pearson’s chi-square test or, when necessary, Fisher’s exact test. The normality of continuous variables was assessed using the Shapiro-Wilk test, and, given the non-normal distribution, comparisons were conducted using the Mann-Whitney U test, with calculation of effect size (Wilcoxon’s r). In the multiple linear regression analysis, the euploidy rate per cycle was used as the outcome variable. Predictors included treatment type and clinical and laboratory variables from both partners. Model assumptions were evaluated, including absence of multicollinearity (VIF), normality of residuals (Shapiro-Wilk), and independence of errors (Durbin-Watson). Coefficients were reported with 95% CI and p-values, and model fit was assessed using adjusted R². Given the non-inferiority design, inference for the primary comparison (PPOS vs GnRH-ant) was based on a confidence-interval approach rather than superiority testing alone. A non-inferiority margin of -15 percentage points was prespecified for the adjusted difference in euploidy rate (PPOS minus GnRH-ant). This margin was chosen *a priori* to represent the largest reduction in euploidy rate considered clinically acceptable in view of the practical advantages of PPOS in freeze-all/PGT-A cycles, including oral administration and lower cost, while preserving overall clinical utility. This margin was also used for sample size planning. All analyses were performed using R version 4.4.1 (RStudio), with a significance level of 5%. Graphs were generated using the ggplot2 package (36, 37).

## Results

A total of 297 treatment cycles were selected between 2021 and 2022. Of these, 24 were excluded due to missing data in medical records and 5 due to the use of donor gametes. Thus, 268 IVF treatment cycles were included in the final analysis, of which 64.5% (n = 173) were in the PPOS group and 35.5% (n = 95) in the GnRH-ant group. Statistical power for the primary outcome was estimated at 85%.

In the adjusted model, the estimated effect of protocol (PPOS vs. GnRH-ant) on euploidy rate was 3.38 percentage points (95% CI, -14.26 to 16.03). Considering the prespecified non-inferiority margin of -15 percentage points, the lower bound of the confidence interval remained slightly above this threshold. Therefore, the formal criterion for non-inferiority was met according to the study design, although with limited margin and requiring cautious interpretation.

The mean female age at treatment was 38.8 years (SD = 3.09; median = 39) in the PPOS group and 39.7 years (SD = 3.13; median = 40) in the GnRH-ant group. Mean male age was 40.9 years (SD = 6.09; median = 41) in the PPOS group and 41.3 years (SD = 5.07; median = 41) in the GnRH-ant group. Age was also analyzed categorically (<35 years vs. ≥35 years), with similar distribution between groups (p = 0.75), indicating no relevant difference in age category distribution.

Baseline differences were observed between groups. Endometriosis was less frequent in the GnRH-ant group (11%, n = 10) than in the PPOS group (30%, n = 49; p < 0.001), although endometriosis severity did not differ among diagnosed patients (p = 0.185). Uterine factor was more prevalent in the PPOS group (37%, n = 61) than in the GnRH-ant group (20%, n = 18; p = 0.005). Varicocele was also more frequent among partners in the PPOS group (13%, n = 21) than in the GnRH-ant group (5%, n = 4; p = 0.030). Male hormonal factor was reported in 5% (n = 9) of the PPOS group and in no patients in the GnRH-ant group (p = 0.026).

**Table 1** summarizes treatment characteristics, COS parameters, and oocyte development and fertilization outcomes according to treatment group. The number of previous IVF attempts was significantly higher in the GnRH-ant group (p < 0.001). Fertilization rate was also significantly higher in the GnRH-ant group (p = 0.004; effect size = 0.24).

**Table 1:** Distribution and comparison between treatments and COS.

Variable	Group	N	Mean	Median	SD	p-value*	Effect size**
N of *in vitro* fertilization attempts	PPOS	163	1.07	0	1.6	< 0.001	0.27
	GnRH-ant	92	1.61	1	1.37		
Right ovarian antral follicles	PPOS	158	5.03	4	3.61	0.059	0.15
	GnRH-ant	83	3.9	4	2.34		
Left ovarian antral follicles	PPOS	158	5.14	4	3.78	0.189	0.1
	GnRH-ant	83	4.31	4	2.21		
Total antral follicles	PPOS	169	10.24	8	6.73	0.054	0.15
	GnRH-ant	84	8.13	7,5	3.95		
Days of treatment	PPOS	165	10.13	10	1.77	0.673	0.03
	GnRH-ant	92	9.9	10	1.84		
Total medication dose	PPOS	164	2,011.89	2,000	364.65	0.823	0.01
	GnRH-ant	92	2,000	2,000	381.73		
N of follicles larger than 14 mm	PPOS	167	7.45	6	4.89	0.837	0.02
	GnRH-ant	80	6.99	6	4.07		
N of oocytes retrieved	PPOS	172	8.13	7	5.32	0.335	0.07
	GnRH-ant	87	7.48	5	4.33		
N of Metaphase I oocytes	PPOS	170	0.38	0	0.78	0.088	0.1
	GnRH-ant	79	0.3	0	0.82		
N of Metaphase II oocytes	PPOS	170	6.55	6	4.13	0.705	0.03
	GnRH-ant	79	6.27	5	3.66		
N of fertilized Metaphase II oocytes	PPOS	161	5.1	4	3.3	0.06	0.16
	GnRH-ant	69	5.68	5	2.95		
Fertilization rate (%)	PPOS	161	81.95	83.33	18.93	0.004	0.24
	GnRH-ant	59	89.1	100	17.78		

*Mann-Whitney U; **Wilcoxon’s r effect size.

Oocyte development and fertilization parameters by treatment group.

**Figure 1:**
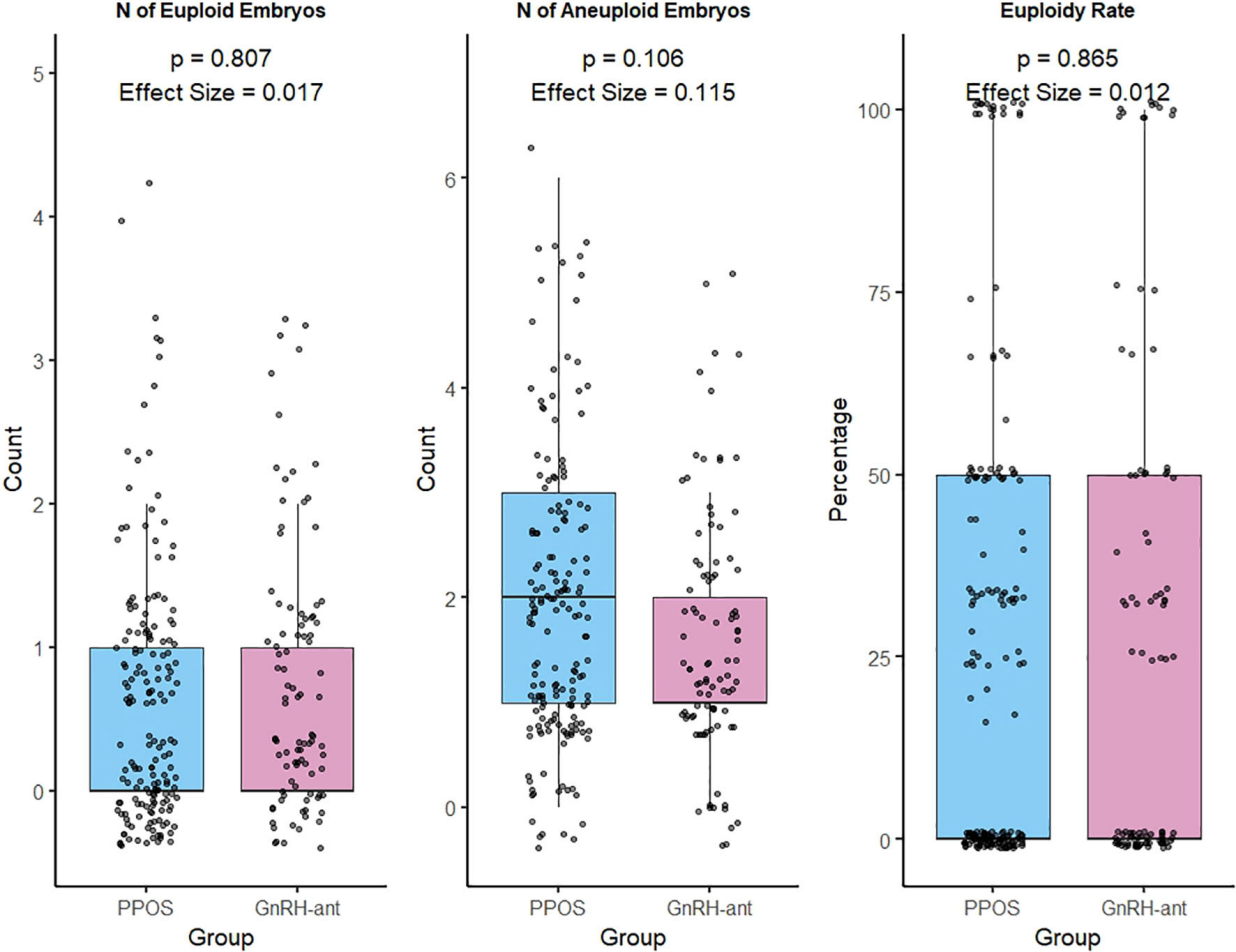
Comparison between treatments according to the number of embryos and euploidy rate.

The mean number of euploid embryos was 0.68 (SD = 0.86) in the PPOS group and 0.72 (SD = 0.90) in the GnRH-ant group, with no statistically significant difference between groups (p = 0.807; effect size = 0.02). The mean euploidy rate was 25.9% (SD = 32.4) in the PPOS group and 27.5% (SD = 34.8) in the GnRH-ant group, also with no significant between-group difference (p = 0.865; effect size = 0.01) (**Figure 1**).

**Figure 2:**
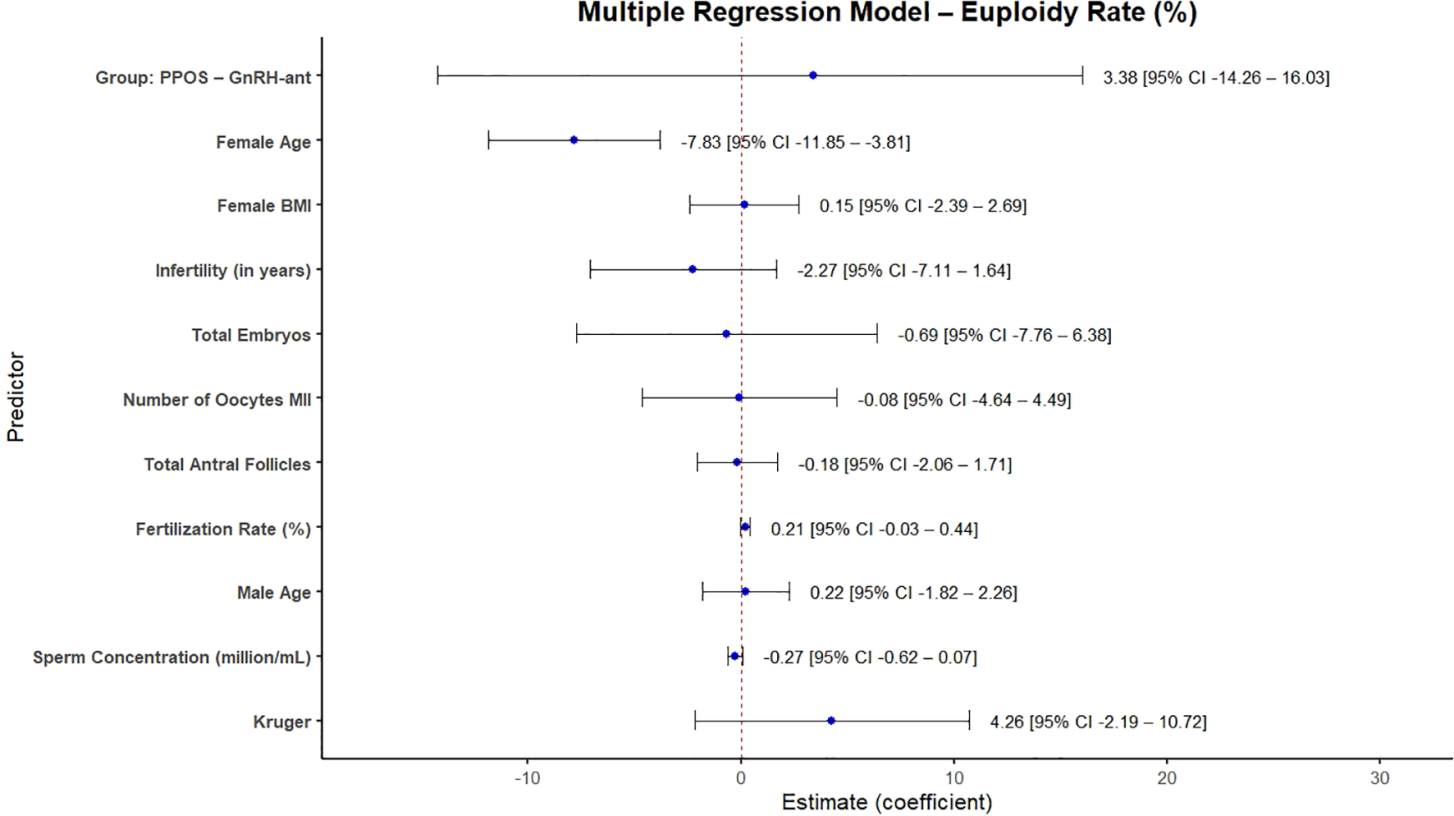
Linear regression model for euploidy rate.

To further explore the association between stimulation protocol and euploidy rate, a multiple linear regression model was fitted including relevant clinical and laboratory covariates (**Figure 2**). In this adjusted model, stimulation protocol was not significantly associated with euploidy rate (estimated coefficient = 3.38; 95% CI: -14.26 to 16.03). Among all predictors evaluated, only female age at treatment remained significantly associated with euploidy rate, with a negative effect (estimated coefficient = -7.83; 95% CI: -11.85 to -3.81). No significant associations were observed for female BMI, infertility duration, total number of embryos, number of MII oocytes, AFC, fertilization rate, male age, sperm concentration, or sperm morphology (Kruger).

As a sensitivity analysis, we fitted a binomial generalized linear model using the same covariates included in the original linear regression model, with the number of euploid embryos per cycle as the dependent variable and the total number of analyzed embryos as the binomial denominator (**Supplementary Table S1**). In this alternative model, stimulation protocol was not significantly associated with euploidy outcome (PPOS vs. GnRH-ant: estimate = -1.390, p = 0.0618), whereas female age remained significantly and negatively associated with euploidy (estimate = -0.496, p = 0.0046). No other covariates reached statistical significance.

## Discussion

The euploidy rate in the PPOS group was 25.9% (SD = 32.4), compared to 27.5% (SD = 34.8) in the GnRH-ant group, with no statistically significant difference between them (p = 0.865, effect size = 0.01), supporting the absence of a large observed difference in this cohort. In this study, a statistical power of 85% was achieved for the primary outcome, indicating that the sample size was adequate to detect clinically relevant differences (35). Additionally, there was no negative impact on ovarian response, embryo quality, or the mean number of euploid embryos. The similarity between protocols regarding treatment duration, gonadotropin consumption, number of retrieved oocytes, number of MII oocytes, number of embryos formed, and embryo morphological quality supports the use of PPOS as a viable alternative in freeze-all/PGT-A settings, although direct safety outcomes were not assessed in the present study.

It is important to note that most studies published to date have been conducted in predominantly Chinese populations (17), limiting the generalizability of their findings. Replicating these findings in Latin American and ethnically mixed populations, as done in the present study, contributes to the external validation of this evidence (14). Our findings are consistent with previous studies, such as those by Vidal et al. (38) and Yang et al. (39), which also found no significant differences between PPOS and GnRH-ant protocols in laboratory outcomes or embryonic euploidy rates. Treatment duration and total gonadotropin dose were similar between groups, with no statistically significant difference, in line with Hendrickx et al. (40).

Mei et al. evaluated the impact of the PO protocol on embryo euploidy and reproductive outcomes in a systematic review. Their findings indicated that PO stimulation yielded euploidy rates and reproductive outcomes comparable to those achieved with conventional ovarian stimulation protocols. Nevertheless, the review presented notable limitations, including substantial heterogeneity that precluded meta-analysis and the predominance of Asian cohorts with limited ethnic diversity, thereby restricting the generalizability of the conclusions (41).

In this study, the fertilization rate was significantly higher in the GnRH-ant group (p = 0.004), contrasting with the findings of Vidal et al. (38) However, this difference in fertilization rate did not affect embryo formation or euploidy rate.

The multivariable regression analysis was used as an additional strategy to account for relevant clinical and laboratory covariates. In this model, maternal age remained the only significant predictor of euploidy rate, reinforcing its well-established role in embryo chromosomal competence. Although the adjusted results did not indicate an independent association between stimulation protocol and euploidy rate, this finding should be interpreted as observational rather than causal (42–44).

An additional clinically relevant observation relates to a potential association between maternal BMI and embryo ploidy. The data suggested a trend toward a higher frequency of aneuploid embryos among normal weight patients. However, maternal BMI was not associated with euploidy in the adjusted regression analysis. This finding warrants further targeted research to clarify the underlying mechanisms (45–50).

In the present study, neither male age nor semen parameters had a significant impact on euploidy rates –results consistent with the meta-analysis by Dviri et al. (51) and other previous studies (52–54). These findings suggest that once fertilization is achieved and the blastocyst stage is reached, the influence of sperm quality on embryonic euploidy becomes limited.

Taken together, the present findings support the use of PPOS-based suppression protocols when fresh embryo transfer is not indicated, particularly in freeze-all/PGT-A cycles, in a Brazilian population marked by intense ethnic diversity (13, 14) – thereby expanding the external validity of the available evidence. However, these findings should be interpreted as laboratory-based and observational, rather than as evidence of full clinical equivalence between protocols. Although PPOS may represent a more convenient and potentially lower-cost strategy in some settings, no formal economic analysis was performed in the present study (55). Additionally, this monocentric study design ensured that all patients were treated using the same laboratory procedures, which strengthens the comparative outcomes.

The retrospective design of the present study requires particularly cautious interpretation, because stimulation protocol allocation was not randomized and baseline differences were observed between groups. These imbalances may reflect differences in infertility profile and routine clinical decision-making. These baseline differences may also have influenced the absence of an observed difference between protocols. However, the main outcome of interest in this study was embryo euploidy rate, a laboratory-based endpoint that is less directly affected by uterine conditions and implantation-related factors than clinical pregnancy outcomes. Likewise, some of the baseline variables that differed between groups, such as endometriosis (56) and selected male-factor conditions (53), are not consistently established as direct determinants of embryo euploidy after blastocyst development and PGT-A assessment. Thus, although euploidy was considered an appropriate endpoint for protocol comparison, residual confounding cannot be excluded and the findings should be interpreted within this context (57).

Although the formal non-inferiority criterion was met, the prespecified margin of -15 percentage points is relatively wide for euploidy rate and the lower bound of the confidence interval approached this threshold. Therefore, the conclusion of non-inferiority should be interpreted cautiously. The findings mainly support the absence of a large observed difference between protocols in this cohort, rather than definitive proof of clinical interchangeability under all assumptions.

Another important limitation is the absence of clinical outcomes such as implantation and live birth rates. Although euploidy rate represents a relevant and objective laboratory-based endpoint, it is an intermediate outcome and does not fully capture reproductive success. Therefore, the clinical implications of these findings should be interpreted with caution.

The classification of mosaic embryos as aneuploid should also be considered an important limitation of the present study. The reproductive potential and optimal clinical management of mosaic embryos remain areas of ongoing investigation, and mosaic results are currently recognized as a distinct interpretative category. Therefore, grouping mosaic embryos with aneuploid embryos represents a methodological simplification that may have influenced the estimated euploidy rates. However, this approach reflected the prevailing clinical practice at our center during the study period (2021–2022), when mosaic embryos were generally managed as non-euploid in clinical decision-making (58).

## Conclusions

Ovulation suppression with PPOS showed euploidy outcomes comparable to those observed with GnRH-ant in this cohort. These findings support the use of PPOS as a viable option in freeze-all/PGT-A cycles, particularly in a genetically admixed population, while also contributing to the external validity of the available evidence. However, given the retrospective design, the baseline differences between groups, and the absence of direct assessment of clinical, safety, and economic outcomes, these results should be interpreted with caution. Maternal age was the only factor independently associated with euploidy.

## Data Availability

The original contributions presented in the study are included in the article/**Supplementary Material**. Further inquiries can be directed to the corresponding author.
