# Supplementary material for: Comparison of euploidy rates between progestin-primed ovarian stimulation and GnRH antagonist protocols: a single-center study in a mixed-ethnicity population
Source: Front Endocrinol (Lausanne). 2026 May 7;17:1788942. doi: 10.3389/fendo.2026.1788942 (PMC13189730; doi:10.3389/fendo.2026.1788942)
Supplement: Supplementary file 1 [file DataSheet1.docx]

**Supplementary Materials**

**Materials and methods**

**In both groups, COS was performed using 100 IU of rFSH daily – referred to as the Protocol 100 – initiated on the second or third day of the menstrual cycle, only in patients aged ≤35 years, with no prior ovarian stimulation or surgery, and with a total AFC greater than 15. Daily doses of 150 or 200 IU of rFSH – referred to as Protocol 150 and Protocol 200, respectively – were used in patients with poor response in previous COS cycles, age >35 years, a single ovary, or a history of ovarian surgery.**^29^

**COS cycles were initiated between day 1 and day 4 of the menstrual cycle, during which a conventional two-dimensional transvaginal ultrasound at 7 MHz (Samsung HS30®) was performed.**^30^ **The ultrasound assessed AFC and evaluated the endometrium, which had to measure ≤5 mm, with no follicles larger than 10 mm. The determination of the gonadotropin dose varied based on ovarian reserve assessment, AFC, age, and other clinical factors.**

In both groups, ovulation was triggered according to the attending physician’s clinical judgment, following a standardized institutional protocol. The decision was based on transvaginal ultrasound assessment of follicular development and overall ovarian response. Ovulation triggering was performed when at least one leading follicle reached a mean diameter of ≥17 mm, in the presence of an adequate cohort of growing follicles, as assessed by follicular number and growth dynamics. The value of 22 mm represents the maximum follicular diameter observed at the time of trigger within the study cohort.

Ovulation triggering was performed using either highly purified hCG or a GnRH agonist, according to clinical indication. Trigger with hCG was carried out using a fixed dose of 5,000 IU of highly purified injectable lyophilized hCG (Choriomon-M®), without dose adjustment according to body mass index. In cycles using GnRH agonist trigger, ovulation was induced with 0.3 mg of triptorelin (three ampoules of Gonapeptyl Daily® 0.1 mg). In selected cases, a dual trigger strategy was adopted, consisting of 5,000 IU of hCG combined with 0.2 mg of triptorelin (two ampoules of Gonapeptyl Daily® 0.1 mg), when a previous cycle showed a maturation response below 50%, or in patients with poor prognosis.^33^ **Oocyte retrieval was performed 35 hours later**, and the oocytes were classified as MII (metaphase II) or MI (metaphase I). All cycles included in the study were performed using ejaculated sperm; no surgically retrieved spermatozoa were used.

**All patients selected for this study underwent embryo biopsy at the blastocyst stage.** Blastocyst-stage embryos were evaluated on day 5 or 6 according to the Gardner grading system.^34^ Biopsy was performed on the trophectoderm, with 5 to 8 cells extracted. The material was sent for whole genome amplification (WGA) and next-generation sequencing (NGS).

**Results**

Regarding **semen parameters**, the **total sperm concentration** was significantly higher in the GnRH-ant group (mean = 162.87 million/mL, SD = 155.90) compared to the PPOS group (mean = 94.49 million/mL, SD = 112.54), with **p = 0.008** and **effect size = 0.32**.

The relationship between clinical and sperm factors and the **genetic outcome of embryos** is presented in **Supplementary Figure S1,** which shows the distribution of Kruger index, female BMI classification, infertility duration, sperm concentration, rapid progressive motility and immotile sperm concentration in relation to embryo **euploidy and aneuploidy**.

**Female BMI classification** showed a **significant association** with embryo euploidy (**p < 0.001**). Most aneuploid embryos were from patients classified as normal weight (84 aneuploid vs. 16 euploid). In contrast, in the overweight category, the distribution was more balanced (34 aneuploid vs. 14 euploid embryos).

Supplementary Figure S1 – Distribution of the Kruger Index, female BMI classification, infertility duration, sperm concentration, rapid progressive motility and immotile sperm concentration in relation to PGT-A results in the euploid and aneuploid groups.


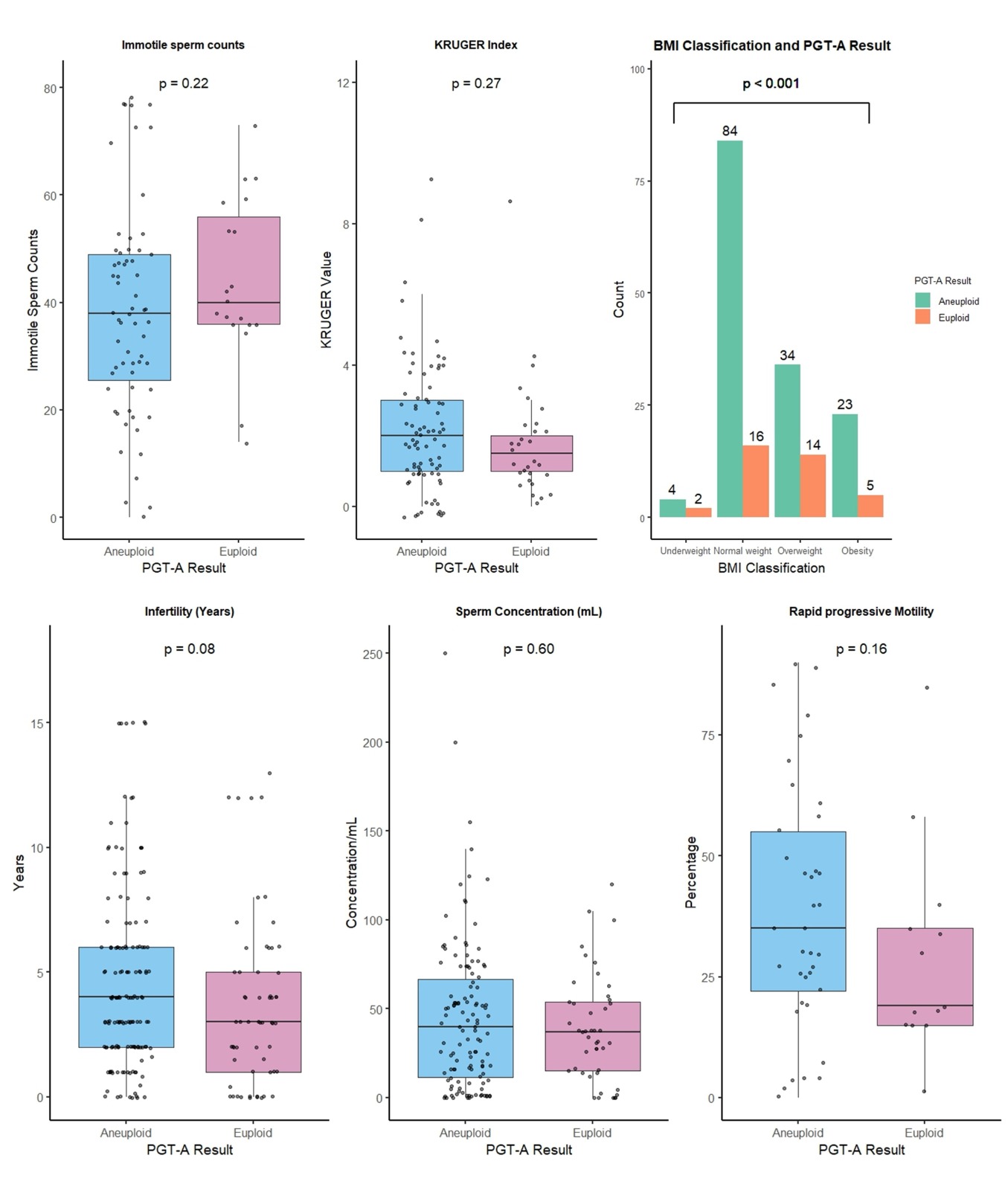


Mann-Whitney U test; Fisher’s exact test
