# Supplementary material for: Comparison of euploidy rates between progestin-primed ovarian stimulation and GnRH antagonist protocols: a single-center study in a mixed-ethnicity population
Source: Front Endocrinol (Lausanne). 2026 May 7;17:1788942. doi: 10.3389/fendo.2026.1788942 (PMC13189730; doi:10.3389/fendo.2026.1788942)
Supplement: Supplementary file 2 [file DataSheet2.docx]

**Supplementary Table S1.** Binomial generalized linear model for the number of euploid embryos per cycle, using the total number of analyzed embryos as the binomial denominator

| **Predictor** | **Estimate (log-odds)** | **Std. Error** | **z value** | **p-value** |
| --- | --- | --- | --- | --- |
| Group (PO vs. GnRH-ant) | -1.390 | 0.744 | -1.868 | 0.0618 |
| Female age | -0.496 | 0.175 | -2.833 | 0.0046 |
| Female BMI | -0.031 | 0.107 | -0.287 | 0.7743 |
| Infertility duration (years) | -0.215 | 0.172 | -1.249 | 0.2115 |
| Total embryos | 0.038 | 0.256 | 0.148 | 0.8826 |
| Number of MII oocytes | -0.078 | 0.127 | -0.612 | 0.5405 |
| Total antral follicles | 0.039 | 0.070 | 0.554 | 0.5795 |
| Fertilization rate (%) | 0.0157 | 0.0172 | 0.911 | 0.3625 |
| Male age | 0.0099 | 0.0843 | 0.118 | 0.9064 |
| Sperm concentration (million/mL) | -0.0107 | 0.0120 | -0.884 | 0.3766 |
| Kruger morphology | 0.145 | 0.263 | 0.553 | 0.5805 |

**Model information:** Null deviance = 55.717 (df = 42); Residual deviance = 30.719 (df = 31); AIC = 77.768.
